# Soft-Tissue Facial Profile Is Associated With Tongue Pressure in Adults: A Cross-Sectional Study

**DOI:** 10.7759/cureus.95200

**Published:** 2025-10-22

**Authors:** Masaki Nakamaru, Norio Aoyama, So Koizumi, Motohiro Komaki, Tetsutaro Yamaguchi

**Affiliations:** 1 Department of Orthodontics, School of Dentistry, Kanagawa Dental University, Yokosuka, JPN; 2 Department of Education Planning, School of Dentistry, Kanagawa Dental University, Yokosuka, JPN; 3 Department of Periodontology, School of Dentistry, Kanagawa Dental University, Yokosuka, JPN

**Keywords:** aging, frailty, malocclusion, muscle, orthodontics, tongue

## Abstract

Objective

Oral frailty, including reduced tongue pressure, has been increasingly recognized as a risk factor for systemic frailty in older adults. Although the relationship between skeletal and perioral muscle function and maxillofacial morphology has attracted growing attention in orthodontics, it is insufficiently understood. This study aimed to investigate the associations between sagittal and vertical soft-tissue facial profile and tongue pressure and skeletal muscle mass in adults.

Materials and methods

This cross-sectional study included 75 participants (median age: 69 years; 67% women) from the Kanagawa Dental University Hospital. Lateral facial photographs were used to measure the soft-tissue nasion-subnasale-soft-tissue pogonion (N'-Sn-Pog') angle (sagittal morphology) and soft-tissue nasion-tragion-soft-tissue menton (N'-Tra-Me') angle (vertical morphology). Tongue pressure was assessed using a balloon-type device, and skeletal muscle mass was measured via body composition analysis. Spearman’s correlation and multiple regression analyses, adjusted for age, sex, and height, were performed.

Results

Tongue pressure showed a significant positive correlation with the N'-Sn-Pog' angle and no correlation with the N'-Tra-Me' angle. Multiple regression indicated an independent association between tongue pressure and the N'-Sn-Pog' angle. A positive association between skeletal muscle mass and the N'-Sn-Pog' angle was found in a model excluding sex as a variable.

Conclusion

In middle-aged and older adults, sagittal soft-tissue facial morphology, as indicated by the N'-Sn-Pog' angle, was positively associated with tongue pressure. These findings suggest that a simple and non-invasive assessment based on facial photographs may serve as a potential indicator of reduced tongue pressure.

## Introduction

As the older population increases worldwide, efforts to support the independent living of older adults are promoted, with an emphasis on extending healthy life expectancy [1,2]. In this context, frailty has become an important concept in the health maintenance of older adults. Frailty is a state of increased vulnerability to health deterioration due to decreased reserve capacity and stress tolerance in multiple physiological systems [3-7]. The major risk factors for frailty include poor physical function, poor nutritional status, and reduced social participation [8]. The decline in physical function, an important component of frailty, is defined as a decrease in grip strength, walking speed, and muscle mass [9].

The relationship between bone and skeletal muscle is important in this decline in physical function. Bone mass and skeletal muscle mass decrease significantly in both men and women as they age [10]. The relationship between bone and skeletal muscle has been recognized as a mechanical interaction in which the muscle loads the bone and the bone supports it [11]. The bone grows in response to functional demands, and the magnitude of the applied load and the bone tissue’s response to it are considered to determine structural design and strength [12]. The bone and muscle cells exchange information at the biochemical and molecular levels, suggesting that such information transfer, in addition to mechanical interactions, may influence bone and muscle functions [11]. In addition, recent studies have defined overlapping impairments in dental and oral functions as “oral frailty” and have reported that this may be a risk factor for physical frailty [2,13-16].

The Japanese Society of Geriatric Dentistry has proposed the concept of oral hypofunction for comprehensive evaluation, management, and intervention for oral function in older adults, and its diagnostic criteria include decreased masticatory function and decreased tongue pressure [17]. Balance of the perioral muscles, including the tongue and buccal muscles, is important in maintaining the position of the dentition, and abnormal tongue function can have a significant impact on the development of malocclusion [18]. Thus, indicators related to frailty and oral frailty are considered important in the field of orthodontics. The association between perioral muscles and maxillofacial morphology has been partially demonstrated. The perioral muscles play a crucial role in shaping the facial structures and determining the jaw relationship, which should be considered when planning orthodontic treatment [19]. However, the knowledge on the relationship between the condition of the general and perioral muscles and maxillofacial morphology is limited. In particular, many unresolved aspects exist in the relationship between the sagittal and vertical morphological features of the maxillofacial region, which are important in orthodontics, and the function of the tongue and skeletal muscles.

Within orthodontic practice, further dissemination of orofacial myofunctional therapy is anticipated; however, research-based evidence is needed to support and promote its broader implementation. Since tongue dysfunction is known to influence the development of malocclusion, tongue pressure may be associated with changes in soft-tissue facial morphology. Therefore, the primary aim of this study was to evaluate whether sagittal and vertical soft-tissue facial morphology are associated with tongue pressure, and a secondary aim was to investigate the relationship between skeletal muscle mass and maxillofacial morphology in adults.

## Materials and methods

Study population

Data for this cross-sectional study were obtained from patient medical records at Kanagawa Dental University Hospital in Yokosuka City, Japan. Results based on previous analyses have been published in several articles. [20-23]. Participants who visited the Center for Medical and Dental Collaboration in Kanagawa Dental University Hospital between December 2017 and January 2020 were selected for this study. The inclusion criterion was age ≥20 years. The exclusion criterion was the presence of neuromuscular disorders or conditions associated with motor impairments. This study was approved by the Ethics Committee of the School of Dentistry, Kanagawa Dental University (approval no. 901) and was conducted in accordance with the Declaration of Helsinki, revised in 2013. The purpose and procedures of the study were explained to all participants, and written informed consent was obtained from each participant. This study was conducted and reported in accordance with the Strengthening the Reporting of Observational Studies in Epidemiology (STROBE) guidelines for cross-sectional studies.

Examinations

All examinations were conducted at the Center for Medical and Dental Collaboration, Kanagawa Dental University Hospital. General information, such as age and sex, was collected from medical records. Body composition analysis was performed using an analyzer (InBody 460; InBody, Tokyo, Japan) to record body indices such as weight and muscle mass. Tongue pressure was measured using a balloon-type tongue pressure device (TPM-01; JMS Corporation, Hiroshima, Japan). In accordance with the manufacturer's instructions, the balloon was placed between the tongue and the anterior part of the hard palate, and participants were instructed to compress the balloon with their tongue three times. The highest value among the three trials was recorded as the maximum tongue pressure.

Photographic soft-tissue profile analysis

Lateral facial photographs of the right side of each participant’s face were recorded following a standardized protocol [24]. During photography, the participants were instructed to remove their glasses, ensure that the forehead and ears were completely exposed, maintain a relaxed position of the lips and mandible, and look at a distance while holding the natural head position. The camera was aligned with the participant’s eye level during image capture. No special procedures were applied to the participants’ faces, such as head fixation or pre-marking of anatomical landmarks. In accordance with these routine procedures, all photographs were taken using a digital camera (EOS 800D; Canon Inc., Tokyo, Japan) equipped with a lens (DCC29-LV/GP3; Sonic Techno Co., Ltd., Tokyo, Japan). The distance between the subject and the camera was set at 1 meter.

**Figure 1:**
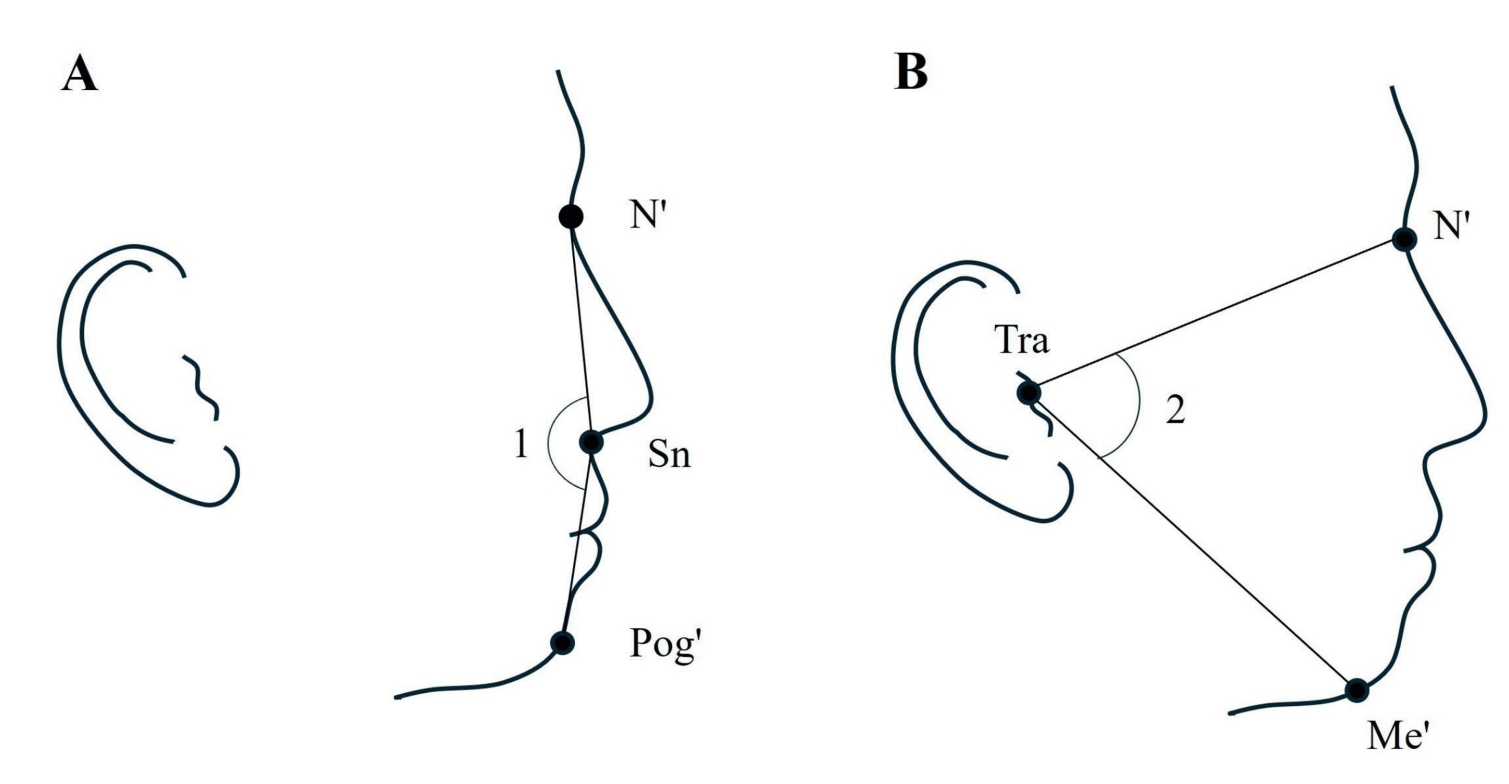
Landmarks and measurements of photographic soft-tissue profile analysis Landmarks: soft-tissue nasion (N'), subnasale (Sn), soft-tissue pogonion (Pog'), soft-tissue menton (Me'), and tragion (Tra). Measurements: 1. Facial convexity angle (N'-Sn-Pog') and 2. N'-Tra-Me'. Figure credit: Masaki Nakamaru

A ring flash was employed as the light source, with the camera settings as follows: shutter speed of 1/125, aperture f/5.6, and ISO 100. The following soft-tissue landmarks were identified for all lateral face photographs (Figure 1): soft-tissue nasion (N'), subnasale (Sn), soft-tissue pogonion (Pog'), soft-tissue menton (Me'), and tragion (Tra). Based on the landmark coordinates, the following angles were measured: sagittal relationship, facial convexity angle (N'-Sn-Pog'), and vertical relationship, N'-Tra-Me' [24-27]. The angles were measured on the photographs using image analysis software (CephaloMetrics AtoZ; YASUNAGA Computer System Co., Inc., Fukui, Japan). Landmark identification and angle measurements were performed by the same evaluator. Landmark identification and angle measurements were performed twice for all lateral facial photographs with a 2-week interval. The intraclass correlation coefficient (ICC) and measurement error were calculated using Dahlberg’s formula [28].

Statistical analysis

Sample size estimation was performed using the G*Power statistical program (version 3.1.9.7; Department of Experimental Psychology, Heinrich Heine University Düsseldorf, Düsseldorf, Germany), with the effect size set at 0.2. According to the power analysis, the minimum required sample size to detect this effect size with 80% power at a 5% significance level was determined to be 65 participants. To ensure sufficient statistical power to detect differences of clinical significance, a small effect size (0.2) was assumed. According to established methodological recommendations, a two-sided test was used for sample size estimation in this study.

The Shapiro-Wilk test was used to test the normality of the data distribution. Numerical data showing a normal distribution are presented as the mean ± standard deviation. Numerical data with skewed distributions are presented as median and interquartile range. The tongue pressure was compared between male and female participants using a t-test. The Wilcoxon test was used to compare differences in skeletal muscle mass between male and female participants. Spearman’s rank correlation coefficient was used to calculate the correlations between variables.

Furthermore, multiple regression analysis was performed with tongue pressure as the dependent variable and age, sex, height, N'-Sn-Pog' angle, and N'-Tra-Me' angle as independent variables to examine the influence of these factors on tongue pressure. Sex was coded as a dummy variable, with male set as 0 and female set as 1. Age was treated as a continuous variable. In model 1, the independent variables included age, sex, N'-Sn-Pog' angle, and N'-Tra-Me' angle. In model 2, sex was excluded from model 1 and replaced with height. Because sex and height were strongly correlated, they were not included in the same model to avoid multicollinearity. Using a similar analytical model, muscle mass was examined as the dependent variable to investigate its relationship with age, sex, height, N'-Sn-Pog' angle, and N'-Tra-Me' angle. All statistical analyses were performed using JMP software version 17.2.0 (SAS Institute Inc., Cary, NC, USA). A p-value of <0.05 was considered statistically significant.

## Results

To assess the reliability of the measurements, the same researcher re-measured them on all lateral face photographs at 2-week intervals. Measurement error was calculated using Dahlberg’s formula [28]. The analysis revealed that the ICC for the N'-Sn-Pog' angle measurement was 0.997, with a random error of 0.63°, and the ICC for the N'-Tra-Me' angle measurement was 0.994, with a random error of 0.74°, indicating a very high level of intra-examiner reproducibility.

The participant characteristics are presented in Table 1. In total, 75 participants were included in this study, comprising 25 men and 50 women. The participants ranged in age from 40 to 89 years, with a median age of 69 years. The median skeletal muscle mass of the participants was 37.3 kg (interquartile range: 33.4-44.8 kg). The mean tongue pressure was 32.2 kPa.

**Table 1:** Characteristics of participants Landmarks: soft-tissue nasion (N'), subnasale (Sn), soft-tissue pogonion (Pog'), soft-tissue menton (Me'), and tragion (Tra). Measurements: Facial convexity angle (N'-Sn-Pog'), N'-Tra-Me'. ^1^Age, height, number of teeth, and skeletal muscle mass are shown as median (interquartile range); ^2^Data are shown as mean ± standard deviation in N'-Sn-Pog', N'-Tra-Me', and tongue pressure.

Variable	Measurement
N	75
Female (n, [%])	50 (67%)
Age (years)	69.0 (62.5–74.8)^1^
Height (cm)	157.4 (152.3–164.1)^1^
N'-Sn-Pog' (°)	171.3 ± 5.8^2^
N'-Tra-Me' (°)	63.5 ± 4.7^2^
Tongue pressure (kPa)	32.2 ±7.3^2^
Skeletal muscle mass (kg)	37.3 (33.4–44.8)^1^

**Figure 2:**
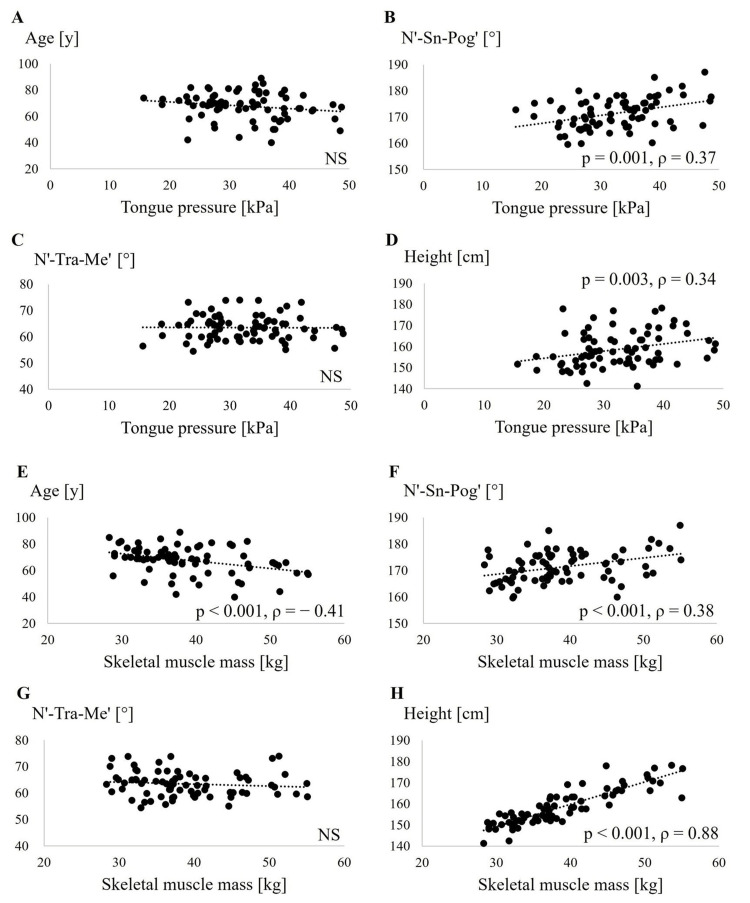
Associations of tongue pressure and skeletal muscle mass with various parameters Age (A), facial convexity angle: N'-Sn-Pog' (B), N'-Tra-Me' (C), and height (D) are compared with tongue pressure. Age (E), facial convexity angle: N'-Sn-Pog' (F), N'-Tra-Me' (G), and height (H) are compared with skeletal muscle mass. N', soft-tissue nasion; Sn, subnasale; Pog', soft-tissue pogonion; Me', soft-tissue menton; Tra, tragion; NS, not significant.

**Figure 3:**
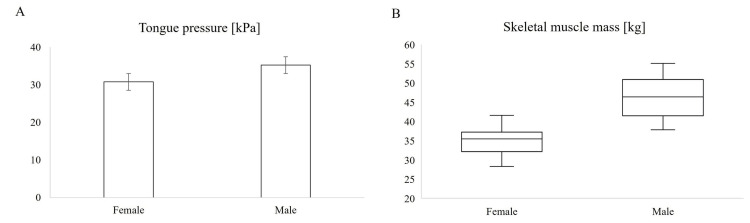
Comparison of tongue pressure and skeletal muscle mass between sexes (A) Mean tongue pressure in male and female participants. Error bars indicate the standard error of the mean; t-test was used for statistical analysis. P = 0.012; (B) Skeletal muscle mass in male and female participants. Box plots indicate the median, interquartile range, and minimum and maximum values; Wilcoxon test was used. P < 0.001.

On assessing the relationship between tongue pressure and age, N'-Sn-Pog' angle, N'-Tra-Me' angle, and height using Spearman’s rank correlation coefficient (Figure 2A-2D), a positive correlation was observed between the N'-Sn-Pog' angle and tongue pressure. In addition, a positive correlation was observed between height and tongue pressure. In contrast, no significant association was found between tongue pressure and age or N'-Tra-Me' angle. As shown in Figure 3, tongue pressure in women was significantly lower than that in men.

The factors related to tongue pressure were evaluated in two models using multiple regression analysis (Table 2). After adjusting for the effects of age and sex in model 1, the N'-Sn-Pog' angle and tongue pressure were positively correlated. Age and N'-Tra-Me' angle showed no association with tongue pressure. After adjusting for the effects of age and height in model 2, the N'-Sn-Pog' angle was significantly associated with tongue pressure. No statistical correlation was found for the effect of height on tongue pressure. No significant association was found for the N'-Tra-Me' angle, even in model 2.

**Table 2:** Results of multiple regression analysis of factors associated with tongue pressure In model 1, the independent variables included age, sex, N'-Sn-Pog' angle, and N'-Tra-Me' angle. In model 2, sex was excluded from model 1 and replaced with height. N', soft-tissue nasion; Sn, subnasale; Pog', soft-tissue pogonion; Me', soft-tissue menton; Tra, tragion; SE, standard error.

Model 1						Model 2					
		Non-standardized						Non-standardized			
Target Variable	Explanatory Variable	B	SE	Standardized β	p	Target Variable	Explanatory Variable	B	SE	Standardized β	p
Tongue pressure (kPa)	Age	-0.081	0.077	-0.104	0.3	Tongue pressure (kPa)	Age	-0.029	0.082	-0.035	0.726
	Sex	-3.16	1.71	-0.184	0.069		Height	0.165	0.103	0.18	0.113
	N'-Sn-Pog'	0.402	0.147	0.247	0.008		N'-Sn-Pog'	0.43	0.145	0.247	0.004
	N'-Tra-Me'	0.12	0.172	0.07	0.488		N'-Tra-Me'	0.123	0.173	0.07	0.477

The relationship between age, N'-Sn-Pog' angle, N'-Tra-Me' angle, and height and skeletal muscle mass was evaluated (Figure 2E-2H). The results showed a positive correlation between the N'-Sn-Pog' angle and height and skeletal muscle mass. A negative correlation was observed between age and skeletal muscle mass. On the other hand, no relationship was found between the N'-Tra-Me' angle and skeletal muscle mass. A comparison by sex revealed that men had significantly greater skeletal muscle mass than women (Figure 3B). Factors related to skeletal muscle mass were evaluated in two models using multiple regression analysis, as was tongue pressure (Table 3). In model 1, adjusted for age and sex, no correlation was found between skeletal muscle mass and N'-Sn-Pog' and N'-Tra-Me' angles. In model 2, adjusted for age and height, we found a positive association between N'-Sn-Pog' angle and skeletal muscle mass.

**Table 3:** Results of multiple regression analysis associated with skeletal muscle mass In model 1, the independent variables included age, sex, N'-Sn-Pog' angle, and N'-Tra-Me' angle. In model 2, sex was excluded from model 1 and replaced with height. N', soft-tissue nasion; Sn, subnasale; Pog', soft-tissue pogonion; Me', soft-tissue menton; Tra, tragion; SE, standard error.

Model 1						Model 2					
		Non-standardized						Non-standardized			
Target Variable	Explanatory Variable	B	SE	Standardized β	p	Target Variable	Explanatory Variable	B	SE	Standardized β	p
Skeletal muscle mass (kg)	Age	-0.236	0.041	-0.317	<0.0001	Skeletal muscle mass (kg)	Age	-0.03	0.041	-0.329	0.469
	Sex	-10.961	0.912	-0.826	<0.0001		Height	0.673	0.051	0.201	<0.0001
	N'-Sn-Pog'	0.094	0.078	0.087	0.235		N'-Sn-Pog'	0.162	0.072	0.087	0.027
	N'-Tra-Me'	-0.068	0.091	-0.053	0.461		N'-Tra-Me'	-0.053	0.085	-0.272	0.534

## Discussion

This study investigated the associations between soft-tissue lateral profile morphology of the maxillofacial region (N'-Sn-Pog' and N'-Tra-Me' angles) and tongue pressure, as well as skeletal muscle mass in adults. The N'-Sn-Pog' angle has been shown to accurately reflect the skeletal Class II pattern, and the N'-Tra-Me' angle is suitable for analyzing hyperdivergent tendencies [24]. The results showed a positive correlation between the N'-Sn-Pog' angle and tongue pressure, while no relationship was found between the N'-Tra-Me' angle and tongue pressure. To the best of our knowledge, the current study is the first to demonstrate an association between oral frailty and lateral facial profile morphology. The results of the association between skeletal muscle mass and N'-Sn-Pog' angle were inconsistent depending on the analytical model, and the results of this study did not provide a clear relationship.

The gold standard for the evaluation of facial skeletal structure is the lateral cephalometric analysis (LCA) of the head by radiography, which was introduced in 1931 [29]. However, LCA requires specialized training and involves radiation exposure. Therefore, in recent years, photographic soft-tissue profile analysis (PSPA), which utilizes lateral facial photographs, has gained popularity as a non-invasive method useful for detecting anteroposterior and vertical skeletal discrepancies. Therefore, in clinical settings and epidemiological screening among older adults, PSPA may be considered to be more practical to use than LCA [24]. In orthodontic clinical practice, the reliability and validity of photographic measurements have already been well established [30,31]. In this study, the facial morphology of the soft-tissue lateral profile was evaluated using PSPA.

The observed positive correlation between the N'-Sn-Pog' angle and tongue pressure suggests that anteroposterior characteristics of maxillofacial morphology may be associated with tongue function. This finding is consistent with that of previous studies reporting an association between low tongue pressure and skeletal class-II malocclusion, supporting the possibility that mandibular retrusion may hinder proper anterior movement of the tongue [18]. In recent years, facial soft tissues have been suggested to respond to functional and orthodontic interventions [32], and these findings support the clinical significance of tongue and perioral functions in soft-tissue adaptations. According to Land and Schoenau [33], bone grows in response to the magnitude of mechanical loading and the reaction of bone tissue. Therefore, reduced tongue activity may suppress mandibular growth and potentially affect maxillofacial morphology. In other words, low tongue pressure may be a contributing factor in the exacerbation of skeletal class-II malocclusion characteristics. However, longitudinal studies involving pediatric patients are needed to further investigate this topic.

The trend toward higher tongue pressure with a larger N'-Sn-Pog' angle suggests that tongue size may affect tongue pressure, as it has been reported in the past that patients with skeletal class-III malocclusion have a larger tongue volume [34] and that tongue size may be a contributing factor to tongue pressure [18]. Tongue pressure is an indicator of the functional strength of the tongue muscles and is closely related to mastication and swallowing function [35]. The findings of this study suggest that the evaluation of tongue function may be important in the orthodontic treatment of skeletal class-II malocclusion. Furthermore, the association between the N'-Sn-Pog' angle and tongue pressure may be an indicator for early detection of oral frailty and intervention. This may serve as a potential indicator for the early detection of oral frailty. Given its simplicity and non-invasive nature, it may have potential for future application in community health screenings and elderly care settings. Future studies are warranted to validate these findings against cephalometric analyses and prospective outcomes to establish their clinical utility.

No significant association was found between the N'-Tra-Me' angle and tongue pressure. Anterior open bite has been reported to potentially result from imbalanced jaw muscle activity, abnormal tongue pressure, or low maximum occlusal force [36,37]. Previous studies have reported that patients with vertical skeletal characteristics, such as a short mandibular ramus height or a clockwise-rotated mandible, tend to have lower tongue pressure [38]. However, no clear association was found in the present study. This may be due to the possibility that factors such as age, sex, and height had a stronger effect on tongue pressure than the N'-Tra-Me' angle. In addition, vertical soft-tissue evaluation using lateral facial photographs may involve a certain degree of error [24,26]. Therefore, further studies considering more detailed factors, such as palatal morphology and hyoid bone position, are needed.

A positive association was suggested between the N'-Sn-Pog' angle and skeletal muscle mass; however, this association disappeared when sex was included as an independent variable. Therefore, based on the results of this study, a clear association between the N'-Sn-Pog' angle and skeletal muscle mass could not be established. Consistent with existing findings that men have more skeletal muscle mass than women [10], the results of this study suggest that skeletal muscle mass is particularly strongly related to sex. Since skeletal muscle mass is likely to be influenced by daily physical activity, these factors need to be considered in future investigations. Although the association between soft-tissue lateral facial morphology and skeletal muscle mass may be explained by biochemical and mechanical interactions between bone and muscle, further investigation is needed to clarify the mechanisms linking facial morphology in the sagittal plane to muscle mass in the trunk and extremities.

Limitations

This study has some limitations. This study has a potential selection bias because the participants were recruited from a hospital-based sample. The tongue pressure measurement protocol did not include control for factors such as time of day, denture/prosthesis use, dental status, or occlusion pattern. It has been shown that facial photographs and cephalometric analysis provide complementary information for the evaluation of facial morphology, while facial morphology may be influenced not only by skeletal factors but also by multiple elements such as soft tissue and muscle function [39]. PSPA has been reported to be a reliable method for screening skeletal class-II and vertical skeletal discrepancies in pediatric patients [24]. Landmarks that are not covered by thick soft tissue have been shown to exhibit a high correlation with lateral cephalometric analysis [27]. However, in middle-aged and older adults, it may be necessary to consider the potential discrepancy between soft tissue and skeletal landmarks, particularly around the Me’ region, due to age-related soft tissue sagging.

In this study, the median age of the participants was 69 years, and many of them were older adults. In this regard, it is necessary to assess the accuracy of PSPA in comparison with cephalometric analysis in middle-aged and older adults, taking factors such as BMI into account in future studies. It has been reported that cone-beam computed tomography, as a 3D imaging modality, provides higher diagnostic accuracy compared to 2D image sets such as panoramic and lateral cephalometric radiographs [40]. Similarly, when evaluating lateral facial photographs, the information obtained is inherently limited because three-dimensional structures are represented in a two-dimensional format. Because soft tissue is susceptible to changes depending on posture and muscle tension, standardization of measurement conditions is essential.

In this study, photographs were taken under standardized conditions to ensure measurement reproducibility; however, this does not guarantee complete consistency. Future studies should compare LCA and PSPA in middle-aged and older adults to evaluate the accuracy of soft-tissue assessment. In addition, observing long-term changes in tongue pressure and skeletal muscle mass may provide a more accurate assessment of the role of soft-tissue lateral facial morphology and tongue function in the progression of oral and general frailty.

## Conclusions

In the present study, a positive correlation was found between the N'-Sn-Pog' angle and tongue pressure. Regarding the association between N'-Sn-Pog' angle and skeletal muscle mass, the results differed among the analytical models. No association was observed between the N'-Tra-Me' angle and either tongue pressure or skeletal muscle mass.
